# Visusverschlechterung nach intravitrealer Injektion von Dexamethason im vitrektomierten Auge

**DOI:** 10.1007/s00347-021-01328-1

**Published:** 2021-03-12

**Authors:** Victoria Reitmeier, Christoph Kern, Maria Poimenidou, Ines M. Lanzl

**Affiliations:** 1 Chiemsee Augen Tagesklinik, Prien am Chiemsee, Deutschland; 2 Klinikum rechts der Isar der Technischen Universität München, München, Deutschland

## Anamnese

Ein 56-jähriger Patient stellte sich zur weiteren Behandlung eines postoperativ persistierenden zystoiden Makulaödems in unserer Praxis vor. Bei epiretinaler Gliose durch eine Uveitis unklarer Ursache wurden beide Augen vor 5 Jahren mit Membranpeeling plus Vitrektomie ophthalmochirurgisch versorgt. Binnen der letzten 3 Jahre erfolgte bereits mehrfach die intravitreale Gabe von VEGF-Inhibitoren durch einen niedergelassenen Kollegen. Hierdurch wurde am linken Auge ein stabiler, trockener Netzhautbefund mit einer bestkorrigierten Sehschärfe (BCVA) von 0,7 dezimal erreicht. Bei ausgeprägterem Befund und schwankender BCVA zwischen 0,3 und 0,5 am rechten Auge fand die letzte intravitreale Injektion mit Aflibercept 8 Wochen vor Erstvorstellung in unserer Praxis statt. Laut Übernahmebericht wurde im Behandlungsverlauf unter Aflibercept das beste therapeutische Ansprechen erreicht.

## Klinischer Befund

**Figure 1:**
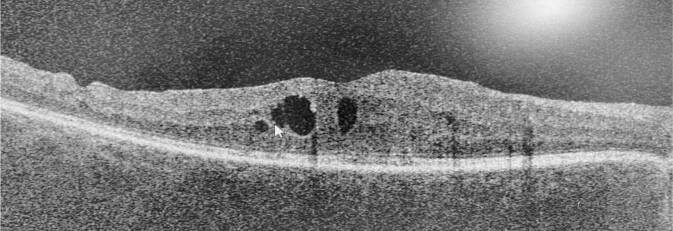

Bei Erstuntersuchung präsentierte sich der Patient mit einer BCVA von 0,6 am rechten und 0,8 am linken Auge. Die vorderen Augenabschnitte waren reizfrei, der Patient pseudophak. Bis auf Pigmentepithelverschiebungen im Bereich der Makula und einem zystoiden Makulaödem war der fundoskopische Befund am rechten Auge unauffällig. In der optischen Kohärenztomographie (OCT) zeigten sich am rechten Auge im Bereich der Fovea intraretinale Zysten im Bereich der äußeren plexiformen Schicht im Sinne eines zystoiden Makulaödems bei einer Netzhautdicke von 350 µm (Abb. 1).

## Behandlungsverlauf

Aufgrund der uveitischen Genese des Makulaödems und damit assoziierter entzündlicher Komponente entschieden wir uns initial für die probatorische Gabe von Nepafenac 3 mg/ml Augentropfen 2‑mal täglich am rechten Auge und Verlaufskontrollen im Abstand von 6 Wochen. Dies entspricht nicht den Leitlinien der DOG zur Behandlung des Makulaödems bei Uveitis [1], jedoch wurde vom Patienten zunächst eine nichtinvasive Therapie gewünscht.

Hierunter verschlechterte sich die BCVA auf 0,2 mit Zunahme der intraretinalen Flüssigkeit in der OCT. Bei schwankendem therapeutischem Ansprechen auf die Gabe von VEGF-Inhibitoren in der Vergangenheit entschieden wir uns deshalb für Dexamethason intravitreal (0,7 mg Dexamethason; Ozurdex, Pharm Allergan GmbH, Frankfurt/M., Deutschland). Die Injektion des Implantats verlief komplikationslos.

Der Patient bemerkte jedoch sofort bei der Injektion eine plötzliche Visusverschlechterung, teilte dies der behandelnden Ärztin nicht mit, wie wir retrospektiv erfahren haben. Den Infektionsängsten zu Beginn der COVID-19-Pandemie geschuldet, erschien er nicht zur vereinbarten postoperativen IVOM-Kontrolle in unserer Praxis.

**Figure 2:**
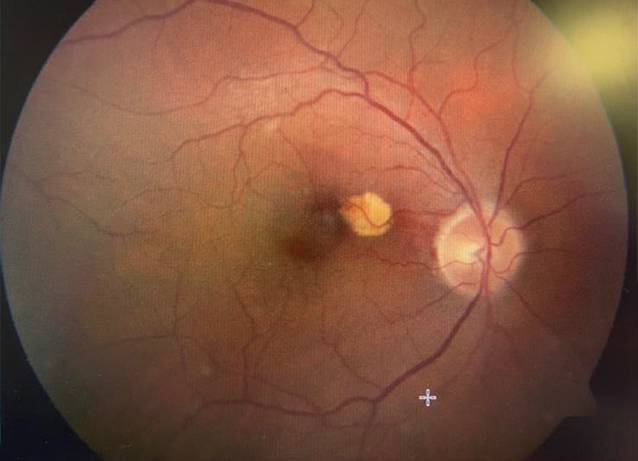

Bei Wiedervorstellung 8 Wochen später wurde eine BCVA von 1/25 Metervisus erreicht, der Augeninnendruck lag bei 31 mmHg. Fundoskopisch zeigten sich im papillomakulären Bereich intraretinale Blutungen und eine gelbliche scharf umschriebene Läsion parafoveal (Abb. 2 und 3).

**Figure 3:**
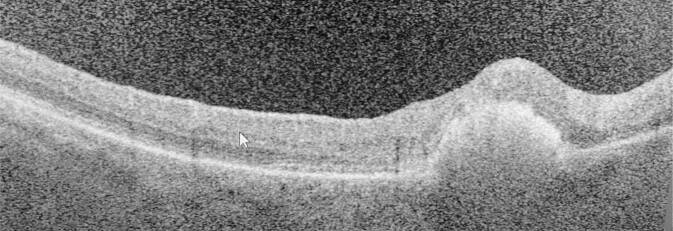

## Wie lautet Ihre Diagnose?

## Therapieverlauf

**Figure 4:**
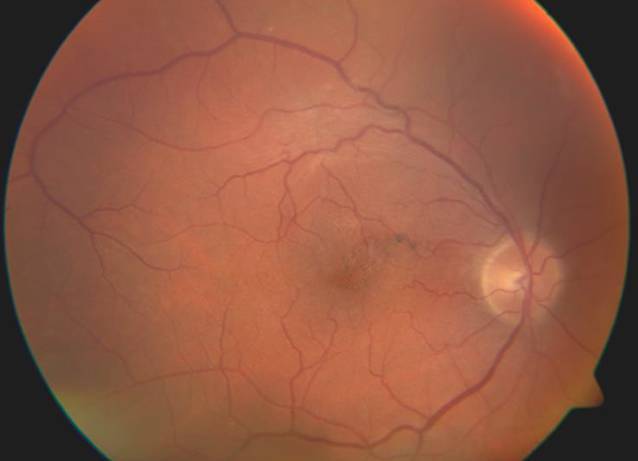

Zur Therapie verordneten wir am rechten Auge topisch Nepafenac 3 mg/ml Augentropfen 2‑mal täglich und Dorzolamid 20 mg/ml /Timolol 5 mg/ml Augentropfen 2‑mal täglich sowie oral 90 mg Celecoxib 1‑mal täglich. Hierunter wurde im weiteren Behandlungsverlauf eine BCVA von 0,16 unter fundoskopischer Remission der Blutung und Resorption der Fibrinmassen erreicht (Abb. 4).

**Figure 5:**
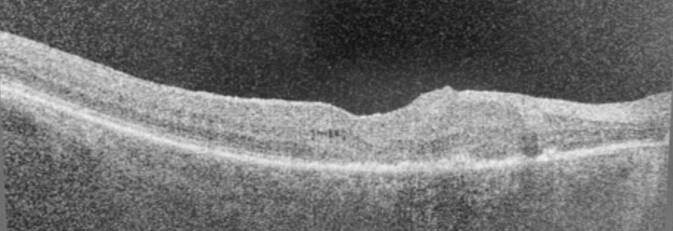

Bei Rückgang der subretinalen soliden Pigmentepithelabhebung blieb in der OCT ein Verlust der äußeren Netzhautschichten, vornehmlich der ellipsoiden Zone zurück (Abb. 5). Das linke Auge zeigte im gesamten Behandlungszeitraum einen stabilen Befund klinisch und in der OCT.

## Diskussion

Das persistierende intraretinale zystoide Makulaödem nach vitreoretinaler Chirurgie konfrontiert behandelnde Ärzte oft mit ausbleibenden Visusverbesserungen, jedoch begrenzten therapeutischen Optionen. Bei intraretinalem zystoidem Ödem nach Uveitis ist die Gabe von Ozurdex auch im vitrektomierten Auge eine therapeutische Option mit gutem Sicherheitsprofil und in der Regel komplikationsarmem Behandlungsverlauf [2, 3]. Bisher unauffällige Anti-VEGF-Injektionen und eine Bulbuslänge von 22,3 mm haben uns ex ante keinerlei Hinweise für ein erhöhtes Komplikationsrisiko gegeben. Gemäß den Herstellerangaben wurde der Applikator parallel zum Limbus eingebracht und für die Injektion senkrecht zum Zentrum des Auges Richtung hinterer Augenpol ausgerichtet [3].

Dies führte bei unserem Patienten zu einem traumatischen Netzhautschaden, welcher in der Literatur als eine äußerst seltene Komplikation sowohl bei vitrektomierten als auch in nicht ophthalmochirurgisch versorgten Augen bereits vorbeschrieben wurde [4–7].

Christensen et al. vermuteten in Konsequenz einen verstärkten Einfluss des veränderten Injektionsmediums nach Vitrektomie durch Fehlen kollagener Fasern, Hyaluronsäure und Opticin und stellten unterschiedliche Kräfte bei Injektion von Ozurdex in verschiedene Medien fest [4]. Vorausgegangene komplikationslose Injektionen von Ozurdex im vitrektomierten Auge bei Casati et al. relativieren diese Vermutung und lassen den traumatischen Netzhautschaden als eher schwer quantifizierbar einstufen [5]. Meyer et al. vermaßen die Mündungsgeschwindigkeit des Ozurdex-Implantates beim Verlassen des Injektors mit einer Hochgeschwindigkeitskamera in Glaskörper und Wasser [8]. Ihre Ergebnisse lassen darauf schließen, dass der direkte Impakt auf die Netzhaut in beiden Fällen keinen Schaden auslösen sollte. Die Autoren stellen auch klar, dass die Injektionsgeschwindigkeit des Implantates durch den Druck gesteuert werden kann, mit dem man auf den Abzugsknopf drückt. Deshalb ist es gerade bei bereits vitrektomierten Augen wichtig, dass man den Auslöser langsam herunterdrückt. Darüber hinaus ist als Konsequenz aus unserem Fall der Injektionswinkel Richtung hinterer Pol, wie in der Gebrauchsanweisung beschrieben, im vitrektomierten Auge zu überdenken. Unserer Meinung nach sollte dieser nicht direkt auf den hinteren Pol, sondern leicht nach nasal ausgerichtet werden, um eine Läsion des papillomakulären Bündels, wie in unserem Fall, zu vermeiden. Eine Aufklärung über die Möglichkeit der traumatischen retinalen Läsion sollte immer und v. a. bei vitrektomierten Augen vor der Injektion erfolgen.

**Diagnose:** traumatischer Netzhautschaden nach Injektion eines Dexamethason-Implantates

In der OCT zeigte sich die fundoskopische Läsion als umschriebene fibrosierte Abhebung. Bei zentraler Netzhautdicke von 240 µm war nun eine Atrophie der vornehmlich äußeren Netzhautschichten mit komplettem Verlust der ellipsoiden Zone zu erkennen. In Zusammenschau der Befunde diagnostizierten wir deshalb einen durch das Dexamethason-Implantat hervorgerufenen traumatischen Netzhautschaden mit konsekutiver, vermutlich traumatisch und durch Blutabbauprodukte bedingter Atrophie im oberen Bereich des hinteren Pols in einem vitrektomierten Auge (Abb. 5).

## Fazit für die Praxis

- Injektionswinkel von Ozurdex im vitrektomierten Auge überdenken.
- Abzugsknopf von Ozurdex bei vitrektomierten Augen langsam durchdrücken.
- Wichtigkeit von Anamnese und postoperativen Druckkontrollen nach IVOM.
- Hinzunahme der Videosprechstunde als postoperative Nachsorge bei COVID-19-bedingten Präsenzeinschränkungen.
